# Acetylation of FOXM1 is essential for its transactivation and tumor growth stimulation

**DOI:** 10.18632/oncotarget.11332

**Published:** 2016-08-17

**Authors:** Cuicui Lv, Ganye Zhao, Xinpei Sun, Pan Wang, Nan Xie, Jianyuan Luo, Tanjun Tong

**Affiliations:** ^1^ Research Center on Aging, Department of Biochemistry and Molecular Biology, Peking University Health Science Center, Beijing, China; ^2^ Center for Medical Genetics, Department of Medical Genetics, Peking University Health Science Center, Beijing, China

**Keywords:** FOXM1, SIRT1, acetylation, cell cycle, tumor

## Abstract

Forkhead box transcription factor M1 (FOXM1) plays crucial roles in a wide array of biological processes, including cell proliferation and differentiation, the cell cycle, and tumorigenesis by regulating the expression of its target genes. Elevated expression of FOXM1 is frequently observed in a multitude of malignancies. Here we show that FOXM1 can be acetylated by p300/CBP at lysines K63, K422, K440, K603 and K614 in vivo. This modification is essential for its transactivation on the target genes. Acetylation of FOXM1 increases during the S phase and remains high throughout the G2 and M phases, when FOXM1 transcriptional activity is required. We find that the acetylation-deficient FOXM1 mutant is less active and exhibits significantly weaker tumorigenic activities compared to wild-type FOXM1. Mechanistically, the acetylation of FOXM1 enhances its transcriptional activity by increasing its DNA binding affinity, protein stability, and phosphorylation sensitivity. In addition, we demonstrate that NAD-dependent histone deacetylase SIRT1 physically binds to and deacetylates FOXM1 in vivo. The deacetylation of FOXM1 by SIRT1 attenuates its transcriptional activity and decreases its protein stability. Together, our findings demonstrate that the reversible acetylation of FOXM1 by p300/CBP and SIRT1 modulates its transactivation function.

## INTRODUCTION

Forkhead Box M1 (FOXM1) belongs to a large family of Forkhead box (Fox) transcription factors that all share an evolutionarily conserved Forkhead/winged helix DNA-binding domain [1-3]. It activates a wide range of target genes by binding to the consensus sequence, TAAACA [4]. FOXM1 plays essential roles in a myriad of biological processes, including cell proliferation and differentiation, cell cycle progression, tumorigenesis, angiogenesis, oxidative stress, inflammation, tissue homeostasis, genomic instability, and metabolism [5-8].

FOXM1 orchestrates the transcription of a wide range of genes that are essential for cell cycle progression and proliferation. FOXM1 is a key regulator for G1/S and G2/M transitions, and M phase progression. It induces the expression of cyclin A2, JNK1, ATF2, Skp2, Cks1, and Cdc25A to promote the G1/S transition [9, 10]. FOXM1 also activates the transcription of a subset/cluster of genes such as cyclin B, Cdc25B, Aurora B, PLK1, Survivin, CENP-A, CENP-B, and CENP-F, which are involved in the G2/M transition, mitotic progression, proper assembly of the mitotic spindles, chromosomal segregation, and cytokinesis [11,12]. Accordingly, depletion of FOXM1 in cells frequently results in diminished S-phase cell population, G2/M arrest, chromosome misalignment, endoreduplication and polyploidization [5, 10-12]. In addition, FOXM1-deficient hepatocytes in mice fail to proliferate and are resistant to the development of hepatic tumors when induced with xenobiotic liver carcinogens [13, 14].

Expression of FOXM1 is restricted in proliferating mammalian cells. Both FOXM1 mRNA and protein levels increase at late G1 phase of the cell cycle and sustained throughout S, G2, and mitosis [15-19]. The transcriptional activity of FOXM1 depends on the activation of the RAS/MAPK pathway and the binding of activated CDK-cyclin complexes to its activation domain. Accordingly, phosphorylation of FOXM1 at Thr596 by cyclin-CDK activates FOXM1 by relieving its autorepression between the N-terminal repression domain and the C-terminal activation domain [20-22], and by mediating recruitment of the p300/CREB binding protein (CBP) [23]. Furthermore, phosphorylation of either Thr 596 or Ser 678 allows for the direct phosphorylation of FOXM1 by Plk1 at G2/M and the subsequent activation of FOXM1 activity. This is required for the expression of Plk1, thereby providing a positive feedback loop leading to further increase in FOXM1 activity [24]. In addition, the phosphorylation of FOXM1 via the Raf/MEK/MAPK pathway also augments FOXM1 transcriptional activity by stimulating its nuclear translocation [25]. It is not at all surprising that FOXM1 activity is so heavily regulated by posttranslational modifications, given its essential role in the regulation of so many biological processes.

FOXM1 plays a critical role in carcinogenesis by promoting cancer initiation, progression, and drug response. It is frequently overexpressed in a broad spectrum of human cancers, including basal-type breast cancer [26], non-Hodgkin's lymphoma [27], malignant peripheral nerve sheath tumors [28], gastric cancer [29], basal cell carcinoma [30], pancreatic cancer [31], prostate cancer [32], cervical cancer [33], head and neck squamous cell carcinoma (SCC) [34], lung cancer [35], colorectal cancer [36], hepatocellular carcinoma (HCC) [37], medulloblastoma [38], malignant mesothelioma [39], and bladder cancer [40]. The critical role of FOXM1 in cancer affirms its significance for therapeutic intervention. Current data suggest that targeting FOXM1 in mono- or combination therapy may have promising therapeutic benefits for the treatment of cancer. Therefore, exploring how FOXM1 is regulated in cells might provide a significant impact on the design of anti-cancer therapeutics.

In this study, we show that FOXM1 is a direct target of CBP/p300 acetylation and SIRT1 deacetylation. During the cell cycle, FOXM1 is initially acetylated at the S phase, hyperacetylated in G2 and M phases, and deacetylated upon the completion of mitosis. Acetylation of FOXM1 by CBP/p300 results in the enhancement of FOXM1-dependent transcription by increasing its DNA binding ability, protein stability, and phosphorylation. On the contrary, SIRT1 induces the inhibition of FOXM1-dependent transcription by specially deacetylating FOXM1. CBP/p300-dependent acetylation and transactivation of FOXM1 promote the transcription of cell cycle genes and thereby contribute to mitotic progression and cell proliferation. Our results demonstrated that acetylation is a critical mechanism in the regulation of FOXM1 activity.

## RESULTS

### FOXM1 Is Acetylated by CBP/p300 in vivo

**Figure 1:**
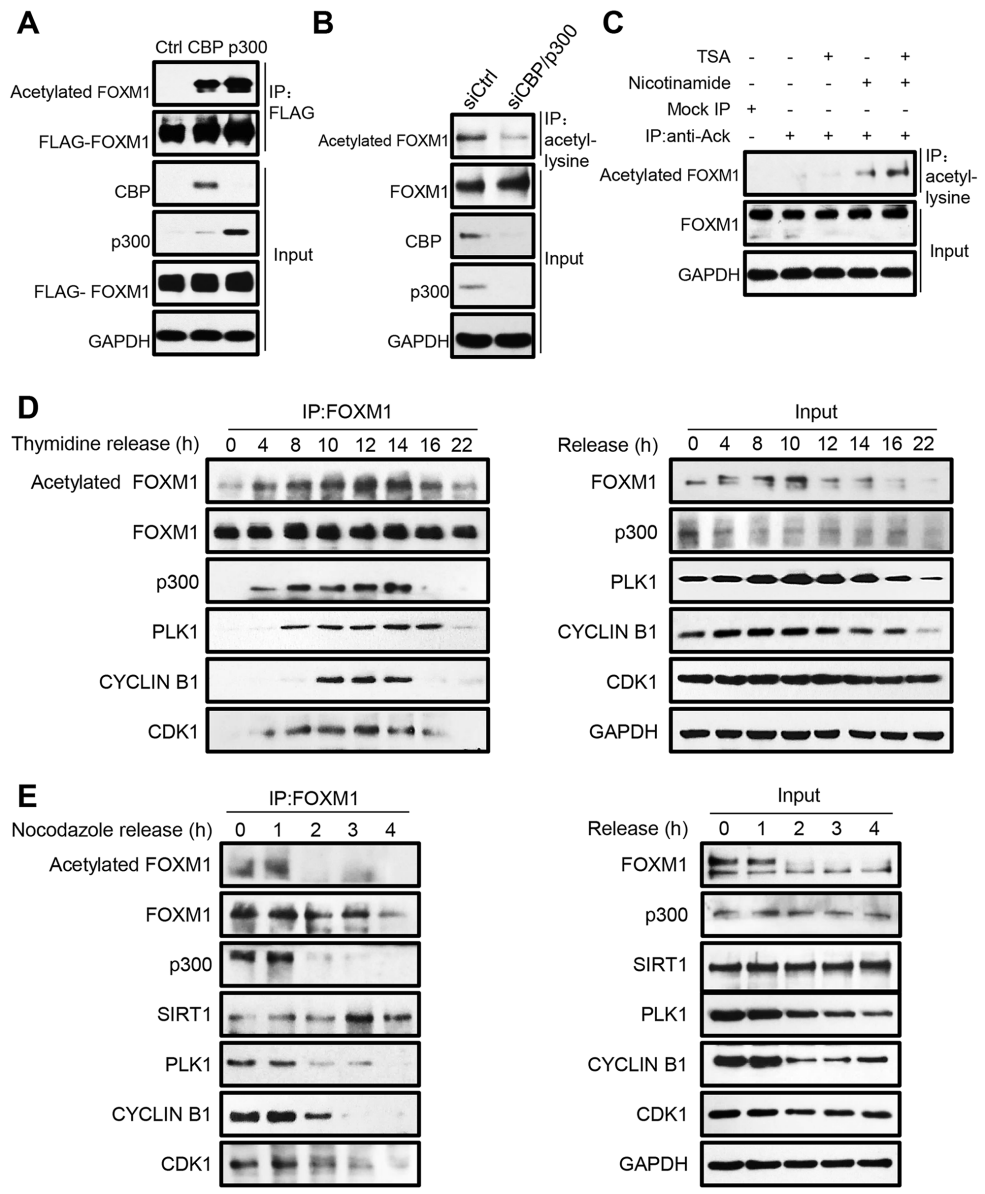
FOXM1 is acetylated by CBP/p300 in vivo **A.** Cell extracts from HEK293T cells co-transfected with FLAG-FOXM1, CBP or p300 were immunoprecipitated with anti-FLAG antibody and analyzed by western blotting with anti-acetylated lysine, and anti-FLAG antibodies. **B.** HEK293T cells were transfected with p300 siRNA and CBP siRNA. The cells were subjected to immunoprecipitation with anti-acetyl-lysine antibody. Immunoprecipitated complexes were used for western blot analysis with anti-FOXM1 antibody. **C.** Whole-cell lysates from HEK293T cells treated with 1 uM TSA or 5 mM nicotinamide alone or in combination were immune-precipitated with control IgG or anti-acetyl-lysine antibody. Endogenous acetylated FOXM1 was analyzed by western blotting with anti-FOXM1 antibody. **D.** U2OS cells were synchronized at the G1/S boundary by double thymidine block, then released into fresh medium, and harvested at the indicated time points (hours). The levels of the indicated proteins were determined by western blotting (right panel). Equal amounts of cell extracts were immunoprecipitated using anti-FOXM1 antibody and analyzed by western blotting with the indicated antibodies (left panel). **E.** U2OS cells were arrested by 300 ng/ml of nocodazole for 16 h. The partially detached arrested cells were shaken-off and washed three times with PBS before being plated to allow re-entry into the cell cycle. Cell extracts were collected at indicated time points and immunoprecipitated with anti-FOXM1 followed by western blot analysis (left panel). Input samples were analyzed by immunoblotting with antibodies as indicated (right panel).

It has been shown that the CBP/p300 acetyltransferases can be recruited to the C-terminal region of FOXM1 and enhance its transcriptional activity at specific stages of the cell cycle [23]. A wide range of non-histone transcription factors have been described to be modified by acetylation recently [41-46], so we investigated whether FOXM1 is acetylated by CBP/p300 in a cellular condition. For this purpose, HEK293T cells were transfected with FLAG-FOXM1 alone or in combination with CBP or p300. After immunoprecipitation (IP) with anti-FLAG antibody followed by western blotting (WB) with anti-acetylated lysines, we observed a prominent increase of acetylated FOXM1 in the cells co-transfected with CBP or p300 (Figure 1A). To further confirm that CBP/p300 was involved in the in vivo acetylation of FOXM1, we examined the endogenous FOXM1 acetylation after depletion of CBP/p300 in cells. As shown in Figure 1B, the depletion of CBP/p300 significantly decreased endogenous FOXM1 acetylation levels. We further examined the endogenous FOXM1 acetylation by treating HEK293T cells with HDAC inhibitor TSA and Sirtuins inhibitor nicotinamide, individually or in combination. As shown in Figure 1C, the acetylation of endogenous FOXM1 was significantly increased when cells were treated with nicotinamide or together with TSA. These results demonstrated that FOXM1 can be acetylated by CBP/p300 in vivo.

To further elucidate how endogenous FOXM1 is acetylated by CBP/p300 during the cell cycle, U2OS cells were synchronized at the G1/S boundary by double thymidine block, and then released into fresh medium to reenter the cell cycle. The cells were collected at the indicated time points for analysis. We first performed flow cytometric analysis to identify the cell cycle stage at each time point. Then, the cell extracts were immunoprecipitated with the FOXM1 antibody, followed by western blot analysis with different antibodies. As shown in Figure 1D (top panel), FOXM1 acetylation levels increased initially in the S phase (hour 4), rose substantially in the early G2 phase (hour 8-10), reached peak levels at the late G2/M phases (hour 12-14), and then decreased when entering the G1 phase (hour 16-22). Parallel with our findings, we observed that the binding between FOXM1 and p300 started at the S phase and mostly occurred during the G2/M phases (Figure 1D, third panel). The FOXM1 protein also displayed its strongest binding to the Cdk1-cyclin B1 complex as well as PLK1 in the G2/M phases (Figure 1D, bottom three panels). Similarly, acetylation of FOXM1 was highly expressed in extracts prepared from cells trapped in G2/M by nocodazole treatment, whereas it dramatically decreased in cells that reentered the G1 phase after 2 h of release from the nocodazole block (Figure 1E, top panel). Consistently, the decreased acetylation levels of FOXM1 were also accompanied with the decreased binding between FOXM1 and p300 and increased binding between FOXM1 and SIRT1 (Figure 1E, third and fourth panel). Taken together, these results demonstrate that FOXM1 is initially acetylated at S phase and reaches its maximum acetylation status at G2 and M phases during the cell cycle.

### FOXM1 is deacetylated by SIRT1

**Figure 2:**
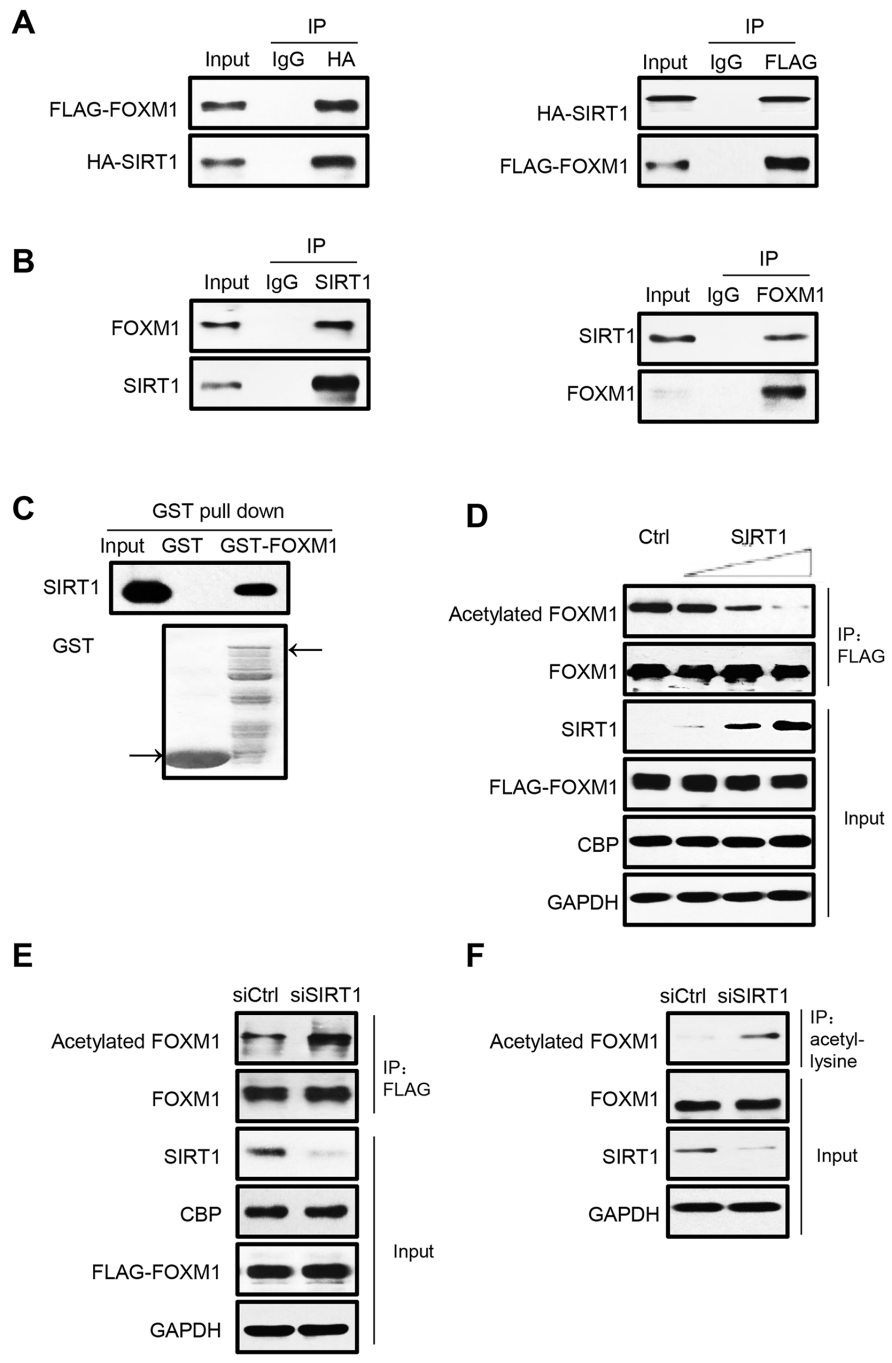
FOXM1 is deacetylated by SIRT1 in vivo **A.** HEK293T cells were transfected with HA-SIRT1 and FLAG-FOXM1. Cell extracts were subjected to immunoprecipitation using IgG as a control or HA or FLAG antibody followed by western blotting with antibodies against HA and FLAG. 5% of cell lysates were used as input. **B.** Cell extracts from HEK293T were immunoprecipitated with SIRT1 or FOXM1 or IgG antibody and analyzed by Western blotting with FOXM1 and SIRT1 antibody. The input lanes represent 5% of the total volume of whole-cell extracts used for the binding assay. **C.** Association between SIRT1 and FOXM1 in vitro. In vitro translated SIRT1 were incubated with GST or GST-FOXM1. CBB staining was used to show the GST-fusion protein levels. **D.** HEK293T cells were cotransfected with plasmids that express FLAG-FOXM1, CBP, and increasing amounts of SIRT1. Whole-cell lysates were immunoprecipitated with anti-FLAG antibody and analyzed by western blotting with anti-acetylated lysine and anti-FOXM1 antibodies. **E.** HEK293T cells were cotransfected with FLAG-FOXM1, CBP, and control siRNA or SIRT1 siRNA. Whole-cell lysates were immunoprecipitated with anti-FLAG antibody and analyzed by western blotting with anti-acetylated lysine, anti-FLAG. **F.** HEK293T cells were transfected with control siRNA or SIRT1 siRNA. Whole-cell lysates were immunoprecipitated with anti-acetyl-lysine antibody, and endogenous acetylated FOXM1 was detected with anti-FOXM1 antibody.

Given the fact that nicotinamide treatment can greatly increase acetylation levels of endogenous FOXM1 (Figure 1C), it is likely that SIRT1 may functionally associate with FOXM1 as its deacetylase. To test this hypothesis, we first investigated if FOXM1 can bind with SIRT1 in mammalian cells. HEK293T cells were transiently transfected with FOXM1 and SIRT1 constructs, and coimmunoprecipitation assays were performed. As expected, FOXM1 can clearly be coimmunoprecipitated by HA-SIRT1 (Figure 2A, left panel), and SIRT1 can also be coimmunoprecipitated by FLAG-FOXM1 (Figure 2A, right panel). Moreover, we also detected that endogenous SIRT1 can bind with endogenous FOXM1 (Figure 2B). GST pull down assay also showed that the in vitro translated SIRT1 associated with GST-FOXM1 but not with GST (Figure 2C), indicating that SIRT1 binds directly to FOXM1 in vitro. To investigate whether SIRT1 is able to deacetylate FOXM1 in cells, we co-transfected increased amounts of SIRT1 together with FLAG-FOXM1 and CBP for the acetylation assay. SIRT1 effectively deacetylated FOXM1 in a dose-dependent manner (Figure 2D). We further examined the effect of RNAi-mediated SIRT1 knockdown on FOXM1 acetylation in HEK293T cells. Acetylation of both ectopically expressed (Figure 2E) and endogenous FOXM1 (Figure 2F) were significantly increased as a result of SIRT1 knockdown. Collectively, we conclude that SIRT1 is a bona fide deacetylase for FOXM1.

### FOXM1 is acetylated at multiple sites

**Figure 3:**
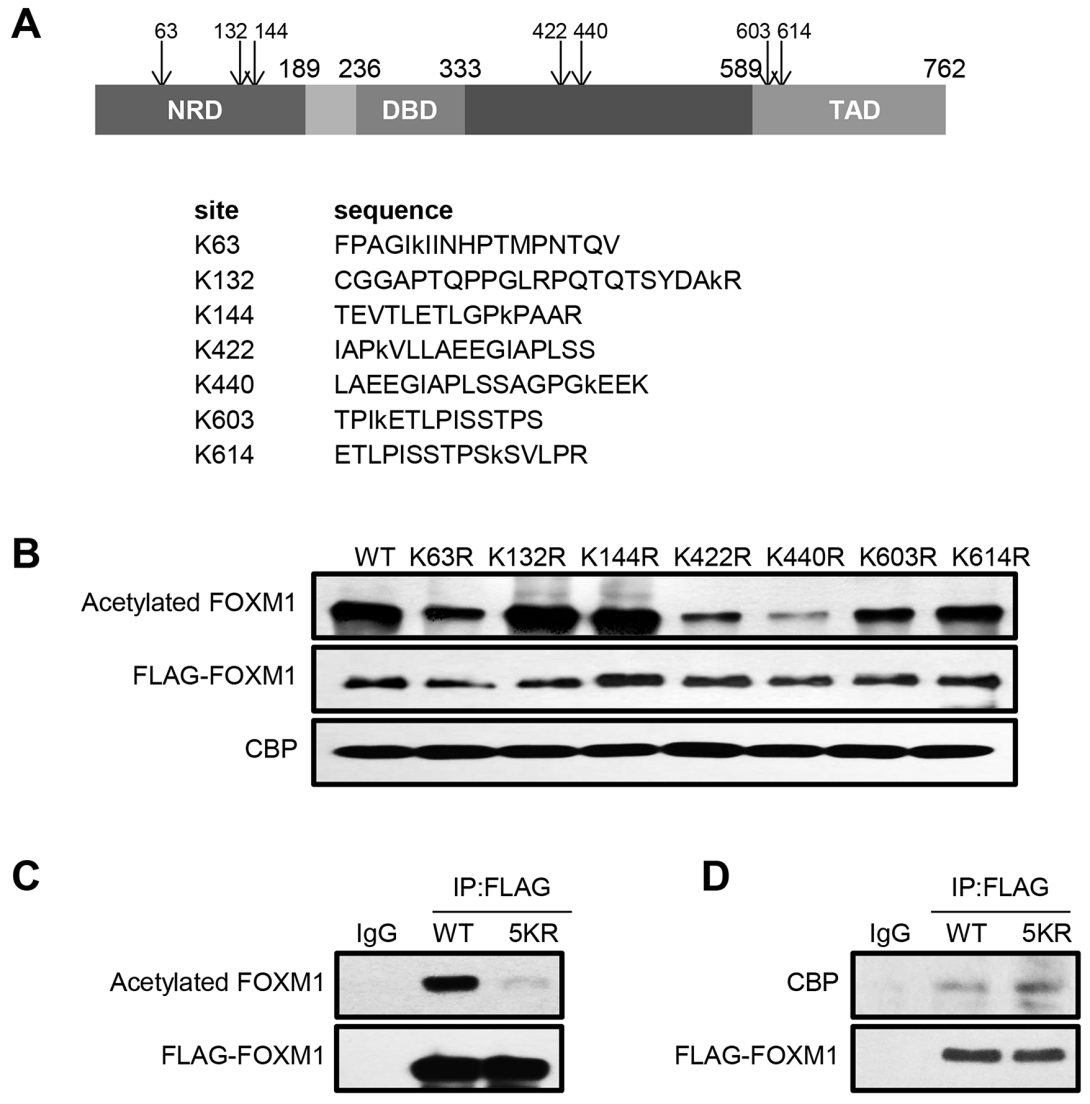
FOXM1 is acetylated at lysines 63, 422, 440, 603 and 614 residues **A.** Schematic showing consensus acetylation sites in FOXM1 identified using mass spectroscopy analysis. **B.** HEK293T cells were cotransfected with CBP and different FLAG tagged FOXM1 constructs. Cell lysates were subjected to immunoprecipitation with anti-FLAG antibody followed by western blotting analysis using anti-acetyl-lysine and anti-FLAG antibodies. **C.** HEK293T cells were transfected with FLAG- FOXM1-WT or FLAG- FOXM1- 5KR. Cell extracts were subjected to immunoprecipitation with anti-FLAG followed by western blotting with anti-FLAG and anti-acetyl-lysine. **D.** HEK293T cells were transfected with FLAG-FOXM1-WT or FLAG-FOXM1-5KR. Cell extracts were subjected to immunoprecipitation with anti-FLAG followed by western blotting with anti-FLAG and anti-p300.

We next sought to identify the potential acetylation sites of FOXM1 by performing mass spectrometry analyses of acetylated FOXM1 proteins. Seven lysines were found to be acetylated by CBP (Figure 3A). To further confirm the major acetylation sites, we generated arginine to lysine substitution mutants at these seven lysines: K63R, K132R, K144R, K422R, K440R, K603R and K614R respectively. HEK293T cells were transfected with either FOXM1-WT or various FOXM1 mutants together with CBP. We observed that – whereas both FOXM1-K132R and FOXM1-K144R constructs were almost acetylated to the same extent as the wild-type FOXM1 protein – FOXM1-K63R, K422R, K440R, K603R, and K614R substitutions were acetylated at a significantly lower level by CBP (Figure 3B). This indicates that K63, K422, K440, K603 and K614 of FOXM1 were the major acetylation sites of FOXM1. Therefore, we simultaneously mutated these five lysines within FOXM1 to produce an acetylation-deficient mutant. As predicted, this mutation (5KR-FOXM1) almost completely abolished the acetylation of FOXM1 in cells overexpressing CBP (Figure 3C). This is a direct result of the mutated lysine rather than a defect in the binding of this mutant to CBP, as FOXM1-5KR bound to CBP at a similar level as the FOXM1-WT (Figure 3D). Altogether, these results indicate that the major acetylation sites of FOXM1 lie at lysines 63, 422, 440, 603 and 614.

### Acetylation enhances FOXM1 transcriptional activity

**Figure 4:**
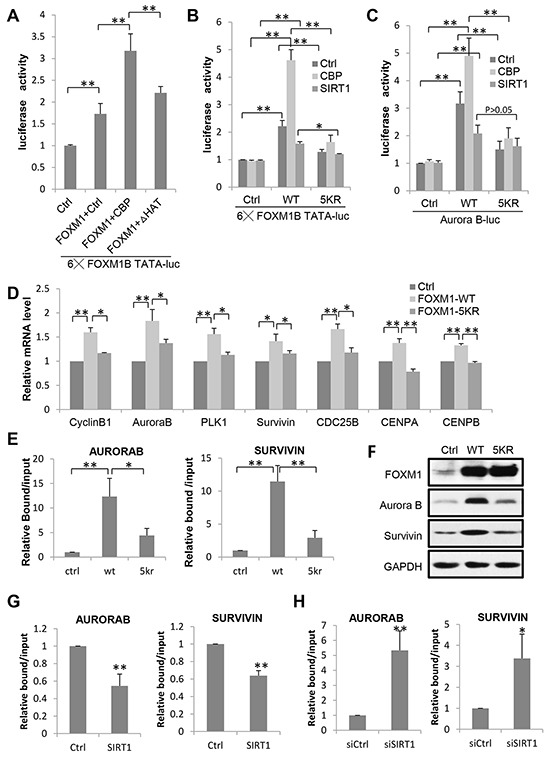
Acetylation Is Required for FOXM1 Transcriptional Activity **A.** Effect of CBP acetyltransferase activity on FOXM1-mediated transcription. U2OS cells were transfected with FOXM1, a luciferase reporter containing six FOXM1 DNA binding domains together with either CBP WT or CBP HAT, and the luciferase activity was measured. **B.** U2OS cells were cotransfected with an empty vector, FLAG-FOXM1 wild-type, or FLAG-FOXM1 5KR, a luciferase reporter containing six FOXM1 DNA binding domains together with CBP or SIRT1. Cells were lysed in reporter lysis buffer 48 h after transfection, and luciferase activity were measured. Results are representative of three independent experiments; the error bars indicate the SD from the average. **C.** U2OS cells were cotransfected with an empty vector, FLAG-FOXM1 wild-type, or FLAG-FOXM1 5KR, a luciferase reporter containing the Aurora B promoter region together with CBP or SIRT1 and the luciferase activity was measured. **D.** Quantitative PCRs were performed on U2OS cells transfected with FLAG-FOXM1 wild-type or FLAG-FOXM1 5KR. The average expression levels of triplicates were normalized for the expression levels of the housekeeping gene GAPDH. **E.** ChIP analysis of FOXM1 enrichment on Aurora B or Survivin promoter was performed in cells transfected with FLAG-FOXM1 wild-type or FLAG-FOXM1 5KR. Data represent the mean ± s.d. for triplicate experiments. **F.** Protein lysates from U2OS cells transfected with an empty vector, FLAG-FOXM1 wild-type or FLAG-FOXM1 5KR were analyzed by western blotting with the antibodies indicated. **G.** ChIP analysis of FOXM1 enrichment on Aurora B or Survivin promoter was performed in cells transfected with ctrl or SIRT1. Data represent the mean ± s.d. for triplicate experiments. **H.** ChIP analysis of FOXM1 enrichment on Aurora B or Survivin promoter was performed in cells transfected with siCtrl or siSIRT1. Data represent the mean ± s.d. for triplicate experiments. *P<0.05; **P<0.01.

To examine the effect of acetylation on FOXM1-mediated transcription, we performed luciferase assays in U2OS cells. Two different luciferase constructs were used, one containing six FOXM1 DNA binding sites (6xFOXM1 DB-luciferase) and one containing the promoter region of the known FOXM1 target gene Aurora B. As shown in Figure 4A, coexpression of wild-type CBP (CBP WT) with FOXM1 potentiated the reporter activity twofold compared to FOXM1 alone. Conversely, the HAT inactive mutant (CBP HAT) had a lesser effect on the CBP-mediated transactivation in the same assay. Thus, CBP served as a transcriptional coactivator of FOXM1 – at least partly through its acetyltransferase activity. To gain a better understanding of the consequence of CBP-dependent acetylation and SIRT1 mediated deacetylation in FOXM1-mediated transcription, we compared the transcriptional activities of wild-type and acetylation-deficient (5KR) FOXM1 on the 6xFOXM1 DB- or Aurora B luciferase reporters. As shown in Figure 4B and 4C, the acetylation-deficient FOXM1 resulted in a significant decrease in FOXM1 transcriptional activity compared to WT-FOXM1 in both reporters. Moreover, when coexpressed with CBP, FOXM1 WT exhibited a marked increase in promoter activity, whereas acetylation-deficient FOXM1 was far less active compared to wild-type FOXM1. In contrast, when coexpressed with SIRT1, FOXM1-mediated transcription activities were slightly repressed. We confirmed these findings by performing quantitative real-time PCR on FOXM1 target genes. Relative mRNA expression levels for Cyclin B1, Aurora kinase B, PLK1, Survivin, Cyclin A2, CDC25B, CENP A, and CENP B were lower in cells transfected with FLAG-FOXM1 5KR compared to cells transfected with FLAG-FOXM1 WT at similar levels (Figures 4D).

We further examined the influence of acetylation on the binding of FOXM1 onto its target genes Aurora B and Survivin promoters by ChIP assays. As shown in Figure 4E, the binding of FOXM1 onto Aurora B/Survivin promoters was greatly impaired in FOXM1-5KR transfected cells, compared to FOXM1-WT. The protein expression levels of these two targets were also examined. The FOXM1-WT transfected cells expressed significantly higher levels of Aurora B and Survivin compared to FOXM1-5KR transfected cells (Figure 4F). Consistent with these results, we found that the binding of FOXM1 onto Aurora B/Survivin promoters was decreased when SIRT1 was overexpressed (Figure 4G) and increased when SIRT1 was knocked down (Figure 4H). These results suggest that the acetylation of FOXM1 enhances its DNA-binding ability and promotes its recruitment to the target genes in vivo. Ultimately, our data demonstrate that the acetylation of FOXM1 by CBP/p300 contributes to the transactivation of FOXM1 to a large extent.

### Acetylation of FOXM1 contributes to its phosphorylation

**Figure 5:**
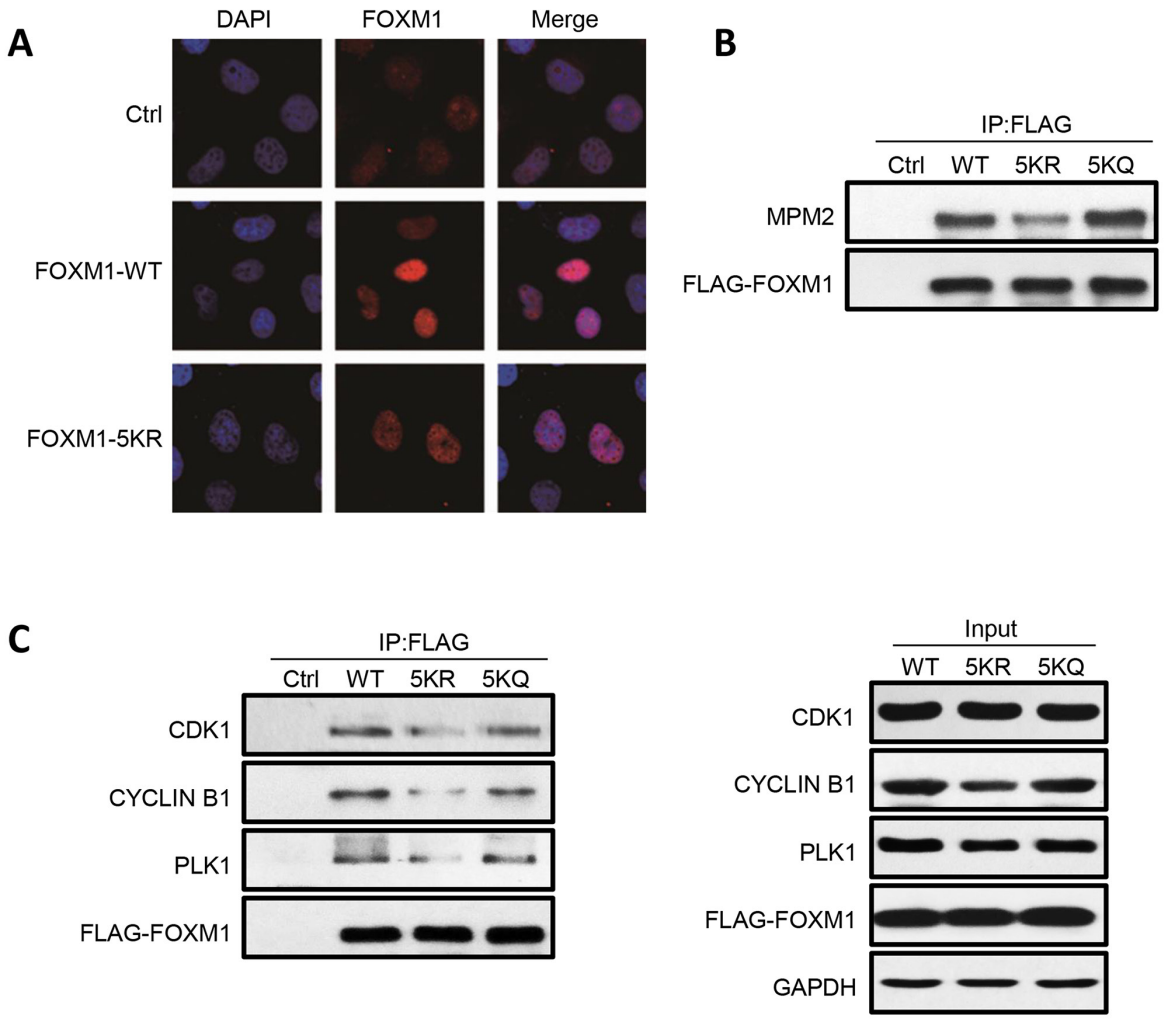
FOXM1 WT, 5KR and 5KQ present differential phosphorylation and interaction with components of the phosphorylation machinery **A.** Fluorescent staining of U2OS cells transfected with empty vector, FOXM1 WT, or FOXM1 5KR using anti-FOXM1. Nuclear DNA was visualized using DAPI staining. **B.** U2OS cells were transfected with FLAG-FOXM1 WT, 5KR or 5KQ, lysed and immunoprecipitated with anti-FLAG followed by analyzing by western blotting with anti-MPM2 antibody. **C.** U2OS cells were transfected with FLAG-FOXM1 WT, 5KR or 5KQ, lysed and immunoprecipitated with anti-FLAG followed by analyzing by western blotting with anti-FOXM1, PLK1, Cyclin B1 and CDK1 antibodies (left panel). Input samples were analyzed by immunoblotting with antibodies as indicated (right panel).

It is well established that FOXM1 becomes progressively phosphorylated throughout cell cycle in ERK- and CDK1-dependent manners, which leads to FOXM1 nuclear localization and activation [20-25]. We first investigated if acetylation of FOXM1 can change its localization; FLAG-FOXM1-WT or FLAG-FOXM1-5KR were transfected into U2OS cells. Immunofluorescent staining demonstrated identical patterns of localization, indicating that the acetylation of FOXM1 had no effect on its nuclear localization (Figure 5A). We further examined whether acetylation has an impact on FOXM1 phosphorylation mediated by CDK1/cyclin B1. U2OS cells were transfected with FLAG-FOXM1-WT, FLAG-FOXM1-5KR, or FLAG-FOXM1-5KQ. Phosphorylated FOXM1 were analyzed by western blot with MPM-2 antibody, an M-phase-specific anti-phospho-Ser/Thr-Pro antibody that recognizes FOXM1. The basal phosphorylation of FOXM1 in the FLAG-FOXM1-5KR mutant was significantly low compared to FOXM1-WT (Figure 5B, lane 3 versus lane 2). However, FOXM1-5KQ – which mimics the hyperacetylated FOXM1 – showed high levels of phosphorylation similar to FOXM1-WT (Figure 5B, lane 4 versus lane 2). This result suggests that there is an interdependency between the acetylation and phosphorylation of FOXM1. To clarify the relevance between FOXM1 acetylation and phosphorylation, we further examined whether acetylation increased the binding between FOXM1 and CDK1/cyclin B1 and PLK1, proteins that reportedly play essential roles in the phosphorylation of FOXM1. A significantly decreased interaction between the FOXM1-5KR mutant and CDK1, cyclin B1, and PLK1 was detected, compared to FOXM1-WT (Figure 5C, lane 3 versus lane 2), whereas the interaction between FOXM1-5KQ and these three proteins showed similar levels to FOXM1-WT (Figure 5C, lane 4 versus lane 2). Altogether, these results demonstrated that FOXM1 acetylation is necessary for the optimal binding between FOXM1 and CDK1, and further contributes to its phosphorylation.

### Acetylation of FOXM1 increases its stability

**Figure 6:**
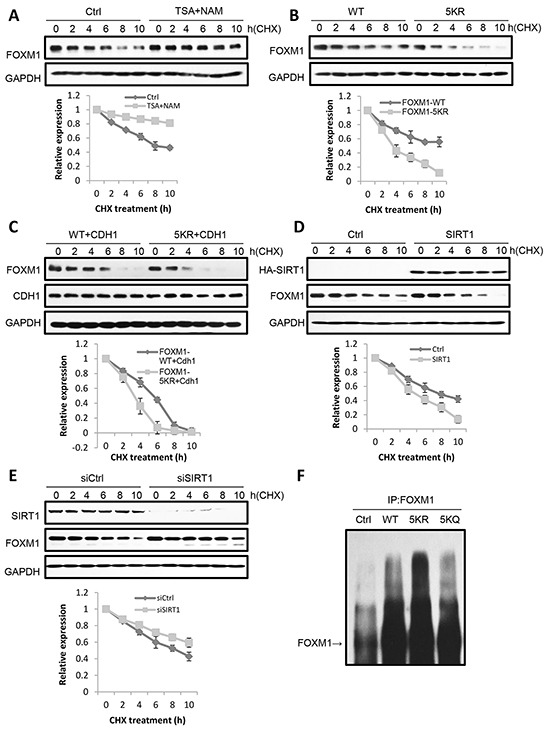
Acetylation increases FOXM1 stability **A.** U2OS cells were treated with 5 mM nicotinamide or 1uM TSA for 16 h. Then cycloheximide (CHX) was added to the cells and cells were collected at different times of CHX treatment. Levels of endogenous FOXM1 were analyzed by western blotting. The amount of FOXM1 was quantitated and represented in a graph. Results are the mean ±s.d. of three independent experiments. **B.** U2OS cells transfected with FLAG-FOXM1 WT or FLAG-FOXM1 5KR were treated with cycloheximide, collected at different times of treatment. FOXM1 levels were analyzed by western blotting. The amount of FLAG- FOXM1 was quantitated and represented in the graph. Results represent the mean ± s.d. of three independent experiments. **C.** U2OS cells cotransfected with FLAG-FOXM1 WT or FLAG-FOXM1 5KR and HA-cdh1 were treated with cycloheximide and collected at different times of treatment. FOXM1 levels were analyzed by western blotting. The amount of FLAG- FOXM1 was quantitated and represented in the graph. Results represent the mean ± s.d. of three independent experiments. **D.** U2OS cells transfected with ctrl or SIRT1 were treated with cycloheximide and then collected at different times after treatment. The levels of SIRT1 and FOXM1 were analyzed by western blotting. The amount of FOXM1 was quantitated and represented in a graph. Results are the mean ±s.d. of three independent experiments. **E.** U2OS cells transfected with siCtrl or siSIRT1 were treated with cycloheximide and then collected at different times after treatment. The levels of SIRT1 and FOXM1 were analyzed by western blotting. The amount of FOXM1 was quantitated and represented in a graph. Results are the mean ±s.d. of three independent experiments. **F.** FOXM1 WT, 5KR and 5KQ present differential ubiquitylation. U2OS cells were cotransfected with HA-ubiquitin and FLAG-FOXM1 WT, FLAG-FOXM1 5KR or FLAG-FOXM1 5KQ and treated with MG132. Cell lysates were subjected to immunoprecipitation with anti-FOXM1 or IgG as a control. The levels of ubiquitylated FOXM1 were determined by western blotting with anti-FOXM1.

Since the acetylation of FOXM1 enhances its transcriptional activity (Figure 4) without changing its localization (Figure 5A) – outside of increasing DNA binding (Figure 4E) – our new inquiry questions whether or not acetylation can participate in the regulation of FOXM1 stability. Following cycloheximide treatment to inhibit protein translation, we observed that the half-life of FOXM1 in TSA and NAM-treated cells was greatly prolonged compared with that observed in non-treated cells (Figure 6A). Consistently, FOXM1-5KR degraded more quickly than FOXM1 (Figure 6B). These results suggested that acetylation was involved in the degradation of FOXM1. Since FOXM1 had been reported to be degraded by the APC/Cdh1 complex during anaphase [41], we wondered whether acetylation had an effect on FOXM1 degradation by the APC/Cdh1 complex. U2OS cells were co-transfected with cdh1 and the WT or 5KR FOXM1, followed by half-life measuring. We found that the co-expression of Cdh1 significantly shortened the half-life of 5KR FOXM1 to a much larger extent than WT FOXM1 following cycloheximide treatment (Figure 6C). Consistently, the overexpression of SIRT1 significantly decreased the half-life of the endogenous FOXM1 (Figure 6D), whereas SIRT1 depletion increased its half-life (Figure 6E). Consistent with previous observations, 5KR-FOXM1 showed higher levels of ubiquitination compared to WT-FOXM1, whereas FOXM1-5KQ was ubiquitinated to an extent similarly to FOXM1-WT (Figure 6F). This supports the idea that acetylated FOXM1 is more resistant to ubiquitination and degradation. Together, we concluded that the acetylation of FOXM1 increases its stability through ubiquitination.

### FOXM1 acetylation promotes cell proliferation and tumor growth

**Figure 7:**
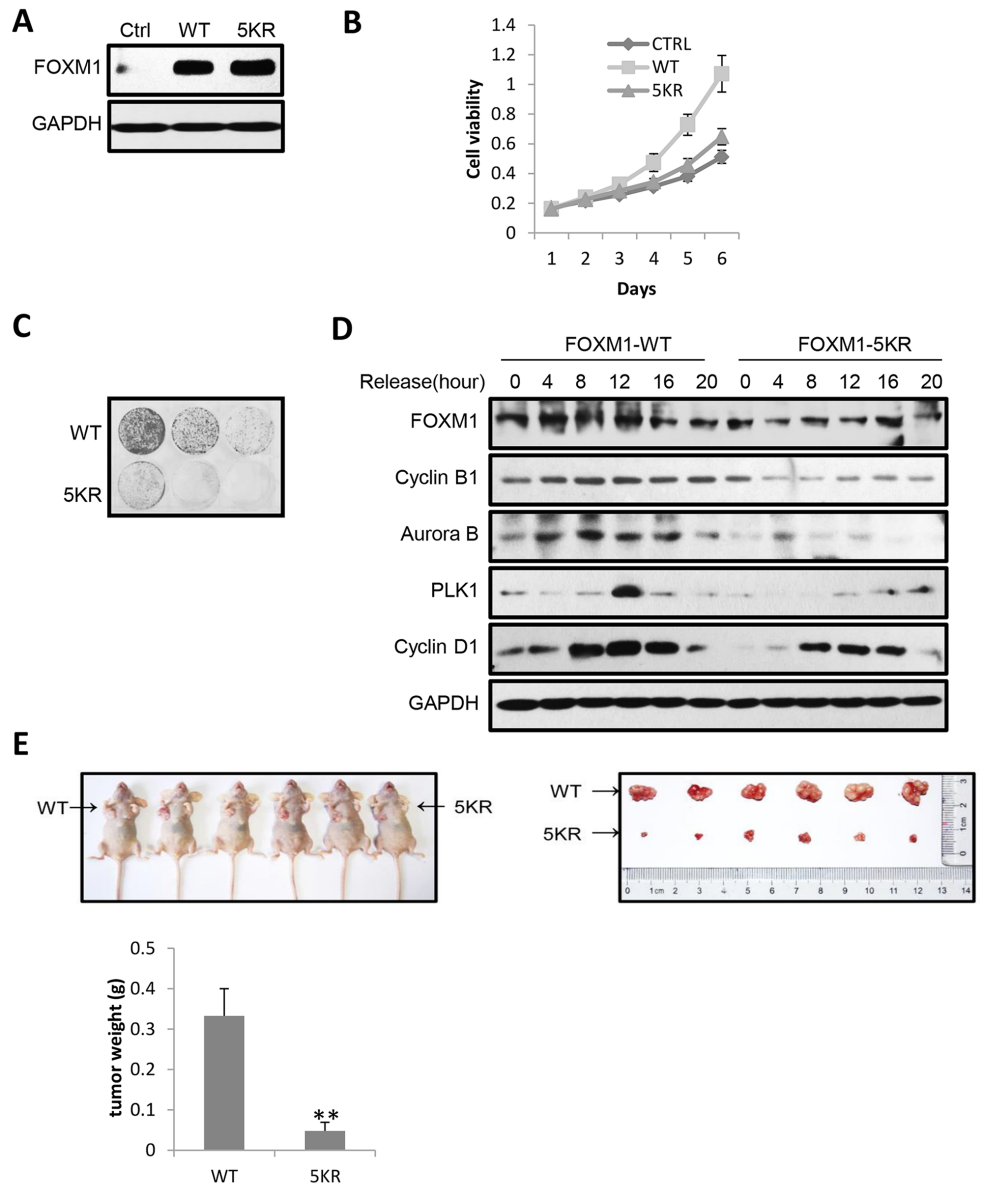
Acetylation of FOXM1 promotes cell proliferation, cell cycle progression and tumorigenic activities **A.** Expression of FOXM1 was determined by western blotting in HeLa cells stably expressing FOXM1-WT or FOXM1-5KR. **B.** The stably expression HeLa cells were cultured for 6 days with their growth curve monitored by MTT assays. **C.** The stably expression HeLa cells were seeded at a density of 1 × 10^3^ or 3 × 10^3^ or 9 × 10^3^ cells per well in 6-well plates and cultured for 15 days. The medium was replaced at 48 h intervals. After 15 days’ culture, the cells were fixed with 4% paraformaldehyde for 15 min at room temperature and then stained with 0.1% crystal violet for 30 min. **D.** HeLa cells stably expressing FOXM1-WT or FOXM1-5KR were synchronized by double thymidine block. Following release from thymidine block, protein lysates were analyzed by western blotting for cell cycle associated cyclins and checkpoint proteins. **E.** HeLa cells stably expressing FOXM1-WT or FOXM1-5KR were subcutaneously injected into the left or right flank of female immune-deficient nude mice respectively. A photograph of the mice is presented. The graph indicates tumor growth in the mice at the end of the experiment. Data represent the mean ± s.d. for the six mice. **P<0.01.

Since FOXM1 was frequently overexpressed in a broad spectrum of human cancers, we further investigated the effect of FOXM1 acetylation on malignant growth potential. We first established HeLa cell lines stably expressing proteins of control, FOXM1-WT, or FOXM1-5KR, and then monitored their phenotypic changes. All three FOXM1 reconstituted cells had similar ectopic FOXM1 expression levels (Figure 7A). The MTT growth curve revealed reduced viability in cells expressing FOXM1-5KR compared to cells expressing FOXM1-WT (Figure 7B). Consistently, crystal violet staining assay indicated that HeLa cells stably expressing FOXM1-5KR displayed markedly suppressed colony-forming ability compared to cells expressing FOXM1-WT (Figure 7C). To further investigate the effect of FOXM1 acetylation in the cell cycle progression, we examined the expression of cell cycle regulatory proteins following double thymidine block and release in HeLa cells stably expressing FOXM1-WT or FOXM1-5KR proteins. Intriguingly, expression of FOXM1-5KR caused a reduction of cyclin B1, Aurora B, PLK1 and cyclin D1 expression compared to FOXM1-WT (Figure 7D). To address the importance of acetylation in regulating the growth of cancer cells, we subcutaneously injected the immune-deficient nude mice with HeLa cells stably expressing FOXM1-WT or FOXM1-5KR. Expression of FOXM1-WT cells grew large tumors within five weeks, whereas a significant inhibition of tumor growth was observed in the same mice injected with FOXM1-5KR expression cells (Figure 7E), which was consistent with the results showed in the MTT growth curve and crystal violet staining assay. Collectively, these data underscore an essential role of acetylation in FOXM1 mediated cell proliferation, cell cycle progression, and tumorigenic activities.

## DISCUSSION

**Figure 8:**
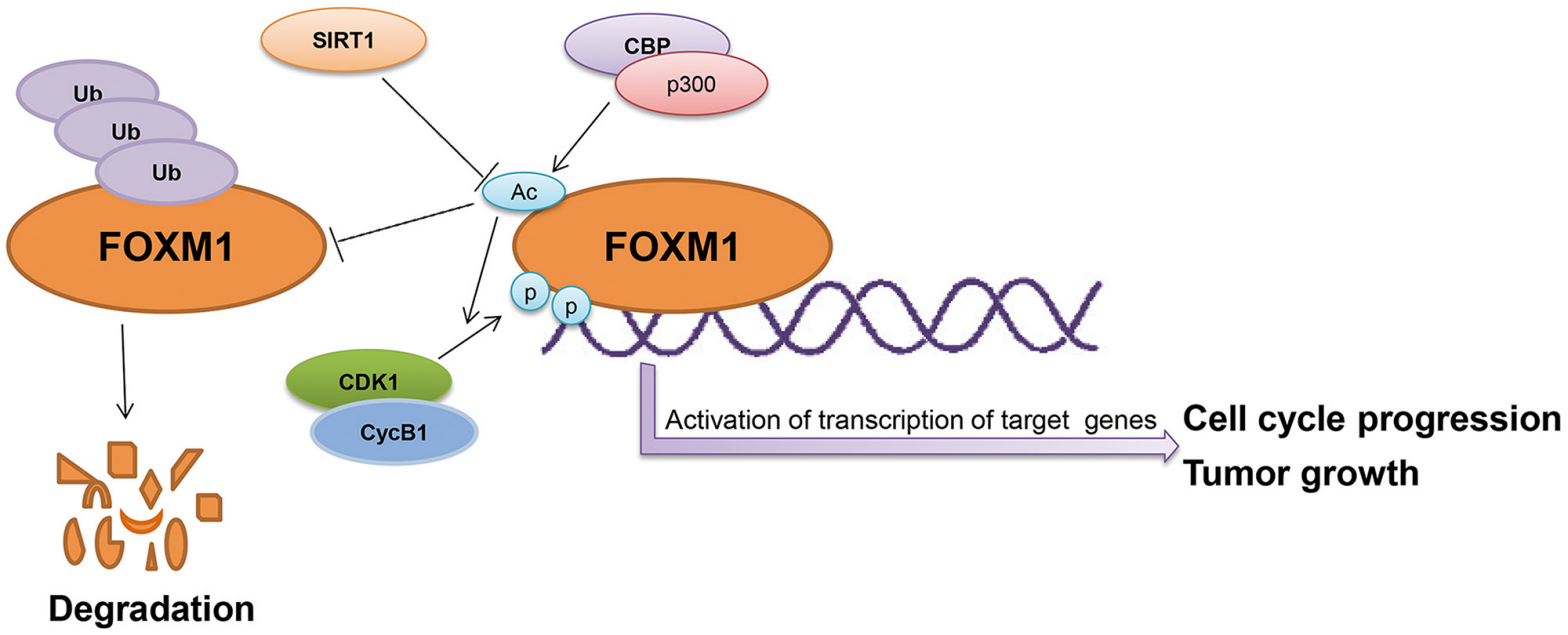
The schematic model depicting acetylation of FOXM1 regulates its functions

The FOXM1 transcription factor plays a significant role in the regulation of a multitude of biological processes, including cell proliferation, oxidative stress, angiogenesis, cancer development, and drug resistance [5-8]. Understanding the regulation of FOXM1 will provide new clues for the identification of reliable and novel therapeutic targets for cancers. In this study, we demonstrated that acetylation and deacetylation play an important role in regulating FOXM1 expression and function. We found that FOXM1 is acetylated by CBP/p300 at lysines 63, 422, 440, 603, and 614. This acetylation is counteracted by SIRT1 during cell cycle progression. Acetylation of FOXM1 potentiates its transactivation function and contributes to cell cycle progression, cell proliferation, and tumorigenesis. Furthermore, we found that SIRT1 serves as a negative cofactor for FOXM1-dependent transcription through its deacetylase activity (Figure 8).

The functional regulation of nonhistone proteins by acetylation/deacetylation has been reported to be mediated through various mechanisms, including changing cellular localization, altering DNA binding properties, affecting protein-protein interaction, modulating enzymatic activities, and influencing protein stability [42-46]. In this study, we observed that the acetylation of FOXM1 has no effect on its nuclear translocation, since both FOXM1-WT and FOXM1-5KR proteins were retained in the nucleus (Figure 5A). Instead, acetylation can enhance FOXM1-dependent transcription by increasing its DNA binding ability (Figure 4E), protein stability (Figure 6), as well as its phosphorylation modification (Figure 5B), indicating that acetylation regulates FOXM1 function in multiple layers (Figure 8).

It is well known that many posttranslational modifications (PTMs) are involved in regulating FOXM1 function, including phosphorylation, ubiquitination, and SUMOylation. For instance, the phosphorylation of FOXM1 is essential in relieving autorepression by the N-terminal Repressor Domain. Phosphorylation of FOXM1 by cyclin A/E-CDK2 [22], cyclin B-CDK1 [23], and PLK1 [24] results in the activation of FOXM1 transcriptional activity, while FOXM1 phosphorylation by Chk2 functions both the transactivation and the enhancement of its stability [47]. Furthermore, phosphorylation of FOXM1 by Raf-MEK-ERK is responsible for its nuclear translocation in the late S phase [25]. It is also reported that SUMOylation of FOXM1 alters its transcriptional activity by mediating its ubiquitination [48] or blocking the dimerization of FOXM1 to relieve FOXM1 autorepression [49]. Our findings on FOXM1 functional regulation by acetylation added another regulating mechanism by PTM. Given that FOXM1 activity is so heavily regulated by PTM, the interplay between PTMs that results in the fine tuning of the biological role of FOXM1 remains to be investigated in the future.

The role of SIRT1 in tumorigenesis is still controversial. In general, SIRT1 was initially considered as an oncogene since it has been found to negatively regulate several tumor suppressors such as p53, FOXO, etc [50, 51]. However, ensuing studies found that SIRT1 also negatively regulates oncogenic proteins such as Survivin, β-catenin, NF-κB, etc [52]. The apparent opposite role of SIRT1 seems contradictory at first, but the seemingly multifaceted functions of SIRT1 make this possible; it seems that the role of SIRT1 as a tumor suppressor or promoter is highly context-specific. In each specific circumstance, the pathway that was dominantly regulated decided the outcome as a tumor suppressor or tumor promoter. In this study, we found that SIRT1 directly binds to and deacetylates FOXM1 (Figure 2), and participates in regulating FOXM1 acetylation status during cell cycle (Figure 1E) and FOXM1 transcriptional activities (Figure 4), indicating that SIRT1 acts as a tumor suppressor in regulating FOXM1 activity. In this regard, the activation of SIRT1 in FOXM1 overexpressed tumors may prove to be a potential weapon in fighting these types of cancers. Furthermore, it is reported that FOXM1 positively regulates the gene transcription of SIRT1 [53]. Our results conclude that SIRT1 induces the inhibition of FOXM1-dependent transcription by specially deacetylating FOXM1. The deacetylation of FOXM1 by SIRT1 attenuates its transcriptional activity, decreases its DNA binding ability, and weakens its protein stability. Therefore, there is a potential regulatory feedback loop between FOXM1 and SIRT1.

The overexpression of FOXM1 has been found in various types of cancers. In this study, we found that the acetylation of FOXM1 can activate its transcriptional activity (Figure 4), increase its stability (Figure 6), and promote cell proliferation (Figure 7B & 7C). Strikingly, the acetylation mutant of FOXM1 (FOXM1-5KR) significantly reduced tumor growth in nude mice (Figure 7E), indicating that FOXM1 acetylation plays an important role in tumor growth. Inhibiting FOXM1 acetylation or increasing its deacetylation activity by the activation of SIRT1 may provide an efficient strategy to fight FOXM1 overexpressed cancers.

## MATERIALS AND METHODS

### Cell culture, transfections and synchronization

HeLa, U2OS, and 293T cells were cultured in Dulbecco's modified Eagle's medium (DMEM, GIBCO BRL, USA) supplemented with 10% fetal bovine serum at 37°C in 5% CO2. Cells were transfected with plasmids using LipofectamineTM 2000 (Invitrogen) following the manufacturer's protocol and then collected at 24 to 48 h after transfection for further analysis. Cells were transfected with siRNA using lipofectamine RNAi MAX according to the manufacturer's instructions (Invitrogen). The short-interfering RNA (siRNA) sequences targeted human p300, CBP or SIRT1 and the transcripts sequences were 5-AACAGAGCAGUCC UGGAUUAG-3, 5-UAGUAACUCUGGCCAUAGC-3 or 5-CGTCTTATCCTCTAGTTCTtt-3.

Cell synchronization was performed by double thymidine block. U2OS cells were arrested at G1/S transition for 17 h with 2.5mM thymidine (Sigma) with a 7-h release interval. And then arrested cells were released into fresh medium to allow cell cycle progression. To isolate mitotic cells, U2OS cells were treated with 300 ng/ml of nocodazole (Sigma) for 16h. The partially detached nocodazole arrested cells were shaken-off and washed three times with phosphate-buffered saline before being plated to allow re-entry into the cell cycle.

### Plasmids

cDNA of wild-type FOXM1 was cloned into pGEX6P-1, pcDNA3.1-FLAG and LPC vectors. cDNA of Cdh1 was cloned into pcDNA3.1. FOXM1 5KR, FOXM1 5KQ and other substitution mutations were generated using the Quick Change mutagenesis kit (Stratagene) according to the manufacturer's instructions and all of them were confirmed by sequencing. SIRT1, p300 and CBP plasmids were described previously (Luo et al., 2001). 6x DB luciferase of FOXM1 reporter was a gift from Prof. Rene H. Medema.

### Western blotting analysis and antibodies

Western blotting assay was performed as described previously (Cao et al, 2011; Zhou et al, 2009). The following antibodies were used in this study: anti-FOXM1 (Santa Cruz, sc-500); anti-acetylated lysine (Cell Signaling Technology, #9681); anti-CDK1 (Abcam, ab133327); anti-MPM-2 (Millipore, 05-368); anti-p300 (Santa Cruz, sc-584); anti-Plk1 (Santa Cruz, sc-17783); anti-CDC25B (Santa Cruz, sc-326); anti-CyclinB1 (Santa Cruz, sc-245); anti-Aurora B (Cell Signaling Technology, 3049S); anti-FLAG (Sigma); anti-Survivin (Cell Signaling Technology, 2808); anti-HA-tag (Cell Signaling Technology); anti-GAPDH (Santa Cruz, sc-47724).

### Coimmunoprecipitation assays

Cells were lysed in lysis buffer (50 mM Tris-HCl, pH 8.0, 150 mM NaCl, 0.5% NP40) for 30 min at 4°C. This was followed by centrifugation at 13,000 rpm for 15 min at 4°C. For immunoprecipitation, 500 ug cell lysates was incubated with 2ug specific antibodies for 10–14 h at 4 °C with end-over-end rotation. Protein A/G-agarose beads were added and the reaction mixtures were further mixed for 2 h at 4 °C. After washed 4 times using the lysis buffer, proteins were eluted from the agarose beads by boiling in 2 x SDS-PAGE loading buffer for 10 min followed by Western blotting.

### Endogenous FOXM1 acetylation assay

HEK293T cells were treated with 1 uM TSA or 5 mM nicotinamide alone or in combination for 6 h before harvest. Whole-cell lysates were immunoprecipitated with anti-acetyl-lysine antibody and protein A/G beads. After being washed with lysis buffer four times, bound acetylated proteins were eluted with 0.1 M glycine (pH 2.5) and were then neutralized with saturated Tris buffer. The eluted proteins were further analyzed by western blot with anti-FOXM1 antibody.

### GST pull-down assays

GST fusion proteins were expressed in *Escherichia coli* strain BL-21 using the pGEX vector system. *In vitro* binding assays were performed by incubating in vitro translated SIRT1with GST-fused proteins immobilized on glutathione-Sepharose in lysis buffer A. After incubation for 4 h at 4°C, the beads were washed four times with the same buffer, and proteins were analyzed by Western blotting.

### Luciferase assays

U2OS cells were grown on 24-well tissue culture plates and transiently transfected with the indicated plasmids. Renilla luciferase plasmid was included to control for the efficiency of transfection, and empty plasmid was added to ensure equal DNA amounts in each transfection. 48 hours after transfection, cells were washed with ice-cold PBS and lysed in 100 μl of Reporter Lysis Buffer (Promega). The firefly and Renilla luciferase activities were monitored using the Dual-Luciferase Reporter Assay System (Promega). The data are shown as the ratio of firefly to Renilla luciferase activity. Luciferase assays were performed in triplicate, and experiments were repeated at least three times.

### Real-time PCR

Total RNA was isolated from cells using RNeasy Mini kit (Qiagen) according to the manufacturer's protocol. Double-stranded cDNA was synthesized using the Star-Script first strand cDNA synthesis kit (GenStar Biosolutions). Real-time quantitative PCR was performed in triplicate using the SYBR Green PCR Master Mix (Invitrogen) on an ABI Prism 7300 Sequence Detector (Applied Biosystems, Foster City, CA) with the expression of GAPDH as the internal control. The sequences of the primers used were provided as follows:

FOXM1, S 5′-GGA GGA AAT GCC ACA CTT AGC G-3′, AS 5′-TAG GAC TTC TTG GGT CTT GGG GTG-3′;

Cyclin-B1, S 5′-TTTCGCCTGAGCCTATTTTG-3′, AS 5′-GCACATCCAGATGTTTCCATT-3′;

Aurora kinase B, S 5′-ATTGCTGACTTCGGCT GGT-3′, AS 5′-GTCCAGGGTGCCACACAT-3′;

Plk1, S 5′- ATC ACC TGC CTG ACC ATT CCA C-3′, AS 5′- TCT CCA AGC CTT TAT TGA GGA CTG-3′;

Survivin, S 5′-TCA AGG ACC ACC GCA TCT CTA-3′, AS 5′-TGA AGC AGA AGA AAC ACT GGG C-3′;

CyclinA2, S 5′-CCT GCA AAC TGC AAA GTT GA-3′, AS 5′- AAA GGC AGC TCC AGC AAT AA-3′;

CDC25B, S 5′-ACG CAC CTA TCC CTG TCT C-3′, AS 5′-CTG GAA GCG TCT GAT GGC AA-3′;

CENPA, S 5′-CTT CCT CCC ATC AAC ACA GTC G-3′, AS 5′-TGC TTC TGC TGC CTC TTG TAG G-3′;

CENPB, S 5′-ATT CAG ACA GTG AGG AAG AGG ACG-3′, AS 5′-CAT CAA TGG GGA AGG AGG TCA G-3′;

GAPDH, S 5′-TCCTCCTGTTTCATCCAAGC-3′, AS 5′-TAGTAGCCGGGCCCTACTTT-3′.

### Chromatin immunoprecipitation assays

U2OS cells were crosslinked with 1% formaldehyde for 10 min at 4°C. After cross-linking, cell extract was prepared in SDS lysis buffer (1% SDS, 10 mmol/l EDTA, 50 mmol/l Tris-HCl, pH 8.1 and protease inhibitors), sonicated, centrifuged and diluted in ChIP dilution buffer (0.01% SDS, 1.1% Triton X-100, 1.2 mmol/l EDTA, 167 mmol/l NaCl, 16.7 mmol/l Tris-HCl, pH 8.1, and protease inhibitors). Chromatin from crosslinked HeLa cells was incubated overnight with anti-FOXM1 or normal rabbit IgG followed by incubation with protein G-Sepharose saturated with salmon sperm DNA. Precipitated DNAs were eluted, decrosslinked and purified followed by quantification with real-time PCR using specific primers for human Aurora B and Survivin promoters region with the results presented as mean+sd for triplicate experiments. The primer sequences for ChIP-PCR analysis were as follows:

Aurora B −856, S 5′-GCA ACG AAA GGT CTA TTG GTG G-3′, and Aurora B −611, AS 5′-TCT AAC TTC TCT GCC CGA TGG AG-3′;

Survivin −1531, S 5′-GGA GGA AGA AGC AGA GAG TGA ATG-3′, and Survivin −1373, AS 5′-CTG GGA TTA CAG ATG TGA GCC AC-3′.

### Growth curves

Cell proliferation was measured by MTT (3-(4, 5-dimethylthiazol-2-yl)-2, 5-diphenyltetrazolium bromide) dye reduction assay. Briefly, cells were seeded into 96-well plates at a density of 1 × 10^3^ cells per well and maintained in culture for days ranging from 1 to 7 days. At the indicated times, the cells were then incubated with 20 μL of MTT (5 mg/ml in 1× PBS; Sigma) for 4 h at 37 °C. After removal of MTT, DMSO was added to the wells. The optical densities of the solutions were measured at 495 nm using an ELISA plate reader. Duplicate measurements were performed on three independent wells at each time point.

### Xenograft model

In the experiment, female BALB/c nude mice (5 weeks of age) were subcutaneously injected with 5 × 10^6^ cells in 200 μL PBS into forelimb armpit. FOXM1-WT and FOXM1-5KR stably expression cells were injected to the left and right flanks, respectively. Tumour growth was monitored every 5 days. The tumour-bearing mice were sacrificed 35 days after inoculation, and the tumours were removed for further study (photographing, weighing). All animal studies were performed in accordance with the guidelines set forth by the Peking University Animal Ethics Committee.

### Immunofluorescence

To detect subcellular localization of FOXM1 by immunocytochemistry, cells were grown on coverslips, fixed in 4% paraformaldehyde/PBS for 15 min at room temperature, washed in PBS and permeabilized with 0.5% Triton X-100/PBS for 15 min followed by blocking with 0.5% BSA in PBS for 1h. Then, the coverslips were incubated with anti-FOXM1 for 12 h at 4 °C. They were then washed in PBS and incubated for 1 h with TRITC -conjugated secondary antibody (Invitrogen). After that coverslips were washed, mounted on glass slides and visualized by fluorescence microscopy. DAPI was used to visualize nuclei.

### Colony formation assay

For the colony formation assay, cells were seeded in the six-well culture plates. After 15 days’ culture, cells were fixed with formaldehyde and stained with crystal violet.

### Statistical analysis

Statistical evaluation of the differential analysis was performed by one way ANOVA and Student's t-test.

## SUPPLEMENTARY MATERIALS FIGURES


