# Supplementary material for: Acetylation of FOXM1 is essential for its transactivation and tumor growth stimulation
Source: Oncotarget. 2016 Aug 17;7(37):60366–82. doi: 10.18632/oncotarget.11332 (PMC5312389; doi:10.18632/oncotarget.11332)
Supplement: Supplementary file 1 [file oncotarget-07-60366-s001.pdf]

Acetylation of FOXM1 is essential for its transactivation and tumor growth stimulation

SUPPLEMENTARY MATERIALS

SUPPLEMENTARY FIGURES

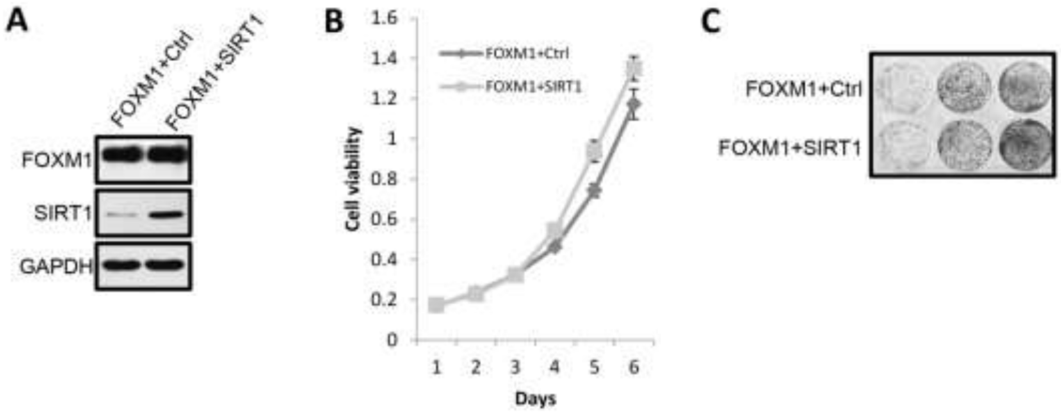

Supplementary Figure S1: SIRT1 promotes cell proliferation and viability in HeLa cells. A. Expression of FOXM1 and SIRT1 was determined by western blotting in HeLa cells stably co-expressing FOXM1 with Ctrl or SIRT1. **B.** The stably expression HeLa cells were cultured for 6 days with their growth curve monitored by MTT assays. **C.** The stably expression HeLa cells were seeded at a density of 1×10^3 or 3×10^3 or 9×10^3 cells per well in 6-well plates and cultured for 15 days. The medium was replaced at 48-h intervals. After 15 days' culture, the cells were fixed with 4% paraformaldehyde for 15 min at room temperature and then stained with 0.1% crystal violet for 30 min.

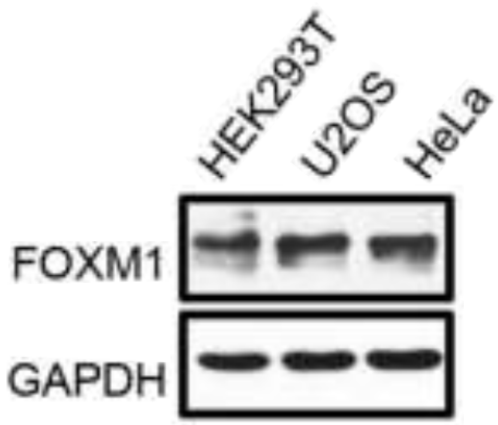

Supplementary Figure S2: The endogenous expression levels of FOXM1 in three cell lines. Cell extracts from HEK293T, U2OS and HeLa cells were analyzed by western blotting with anti-FOXM1 antibody.
